# ERBB3 mediates the PI3K/AKT/mTOR pathway to alter the epithelial‑mesenchymal transition in cervical cancer and predict immunity filtration outcome

**DOI:** 10.3892/etm.2023.11845

**Published:** 2023-02-15

**Authors:** Xiaoyue Yang, Weipei Zhu

**Affiliations:** Department of Obstetrics and Gynecology, The Second Affiliated Hospital of Soochow University, Suzhou, Jiangsu 215000, P.R. China

**Keywords:** human epidermal growth factor receptor 3, epithelial-mesenchymal transition, tumor microenvironment, immunooncology, cervical cancer

## Abstract

Cervical cancer is the fourth most common cancer among women worldwide, and the prognosis of advanced/recurrent cervical cancer remains poor. Metastasis and invasion of this type of cancer are closely associated with the tumor microenvironment. Studying the complex interactions between tumor progression and immune cells or stromal cells can provide new insights into treatment for patients with aggressive tumor, recurrence and drug resistance. In the present study, a bioinformatics method (Gene Expression Profiling Interactive Analysis, differentially expressed genes, Gene Ontology, Kyoto Encyclopedia of Genes and Genomes, protein-protein interactions and survival analysis) was used to explore the mRNA and protein level discrepancy gene signature of ERBB3 via the PI3K/AKT/mTOR pathway from the speculation in immuno-oncology and experimental verification of different cervical cancer cell lines. The high expression of ERBB3 in cervical cancer tissues (especially HPV-positive and adenocarcinoma-related) promoted the activation of the PI3K/AKT/mTOR pathway. The increased expression of MMP9 changed the macrophage infiltration in the tumor microenvironment and affected prognosis of patients with cervical cancer. In conclusion, the present study identified 14 EMT-related genes and 30 genes involved in the PI3K/AKT/mTOR pathway in cervical cancer, and they might provide novel clues for future treatment. The MMP family may be a notable factor associated with tumor cells and immune cells.

## Introduction

Cervical cancer is the most common malignant tumor of the female genital tract worldwide (1). Persistent high-risk human papillomavirus (HPV) oncoproteins E6 and E7 are the main pathogenic factors of cervical cancer (2). At the molecular level, the HPV E6 and E7 proteins directly activate Akt, and this pathway is further stimulated in cervical cancer cells by amplifications and mutations of the PI3K genes. As it has a key role in the control of HPV gene expression and development of cervical cancer, the PI3K/AKT/mammalian target of rapamycin (mTOR) pathway may have potential as a therapeutic target for cervical cancer (3-5).

The tumor microenvironment (TME) contains stromal cells and immune cells that shape cancer development and impact responses to tumor therapy (6). Cancer cell proliferation, angiogenesis and metastasis also contribute to the establishment of an immunosuppressive environment. These factors are associated with tumor progression and poor clinical outcomes (7,8). However, factors that contribute to immunosuppression in the TME are poorly defined.

Epithelial-mesenchymal transition (EMT) plays an important role in tumor development from initiation to metastasis. EMT contributes to the majority of the hallmarks of cancer and continues to be an attractive target for cancer therapy (9). Classical EMT is characterized by the phenotype change of epithelial cells to cells with mesenchymal properties, but EMT is also associated with multiple other molecular processes, including tumor immune evasion (10). Immunosuppression occurs as a direct consequence of the EMT program or develops through some additional, still-uncharacterized signaling channels (11).

The PI3K/AKT/mTOR pathway can promote migration and induce EMT in numerous types of tumors, including cervical cancer (8,12). EMT-related changes in the expression of various receptor tyrosine kinases (RTKs) (13) have been reported, although the proliferation and survival dependence of specific RTKs under different conditions remains to be fully elucidated. The expression of an EGFR family member-ERBB3(14) is associated with the epithelial phenotype of the cell line and the sensitivity to EGFR inhibition. ERBB3 heterodimerizes (15) with additional EGFR family members after stimulation with various ligands, including neuregulin (16). ERBB3 contains multiple binding sites for p85, which is the regulatory subunit of PI3K (17). This allows direct recruitment and activation of PI3K signals via ERBB3(18). Although changes in ERBB3 expression have been observed, the functional consequences of these changes and the relationship with downstream signals after EMT (19) have not been fully described.

These targets involved in cervical cancer are not functionally exclusive; rather, they are intertwined and reciprocal, and together they form intricate TME networks to meet context-specific needs for cellular function. To improve understanding of the correlation between TME and prognosis of cervical cancer, the present study assessed cervical cancer cell lines and tissues to reveal the roles of ERBB3 in the EMT induction of TME harboring immunosuppression, migration and invasion of cervical cancer, and to explore whether PI3K/AKT/mTOR signaling is involved in this process.

The current study intended to explore how ERBB3 mediates the PI3K/AKT/mTOR pathway and changes the tumor immune microenvironment to affect the EMT status of cervical cancer, which may provide further understanding of MMPs involved in immunotherapy.

## Materials and methods

### Data collection and preprocessing

RNAseq data in the transcripts per million (TPM) format from The Cancer Genome Atlas (TCGA) (https://portal.gdc.cancer.gov/) and The Genotype-Tissue Expression (GTEx) (https://gtexportal.org/) were uniformly processed using the Toil process (20). Through extraction of the cervical squamous cell carcinoma and endocervical adenocarcinoma (CESC) data in TCGA and corresponding normal tissue data in GTEx, the 306 cervical tumor samples were classified as the malignant group and the three samples adjacent to cancer from TCGA together with the 10 normal cervix tissues from GTEx were classified as the non-malignant group. The RNAseq data in TPM format were log2 transformed for expression comparison between samples.

### Related hub genes selection

A total of 14 EMT-related genes were selected based on the article ‘Guidelines and definitions for research on epithelial-mesenchymal transition’ written by the EMT International Association (TEMTIA) in 2020(21). When searching for pathway related genes, GeneCards (https://www.genecards.org/) was used; the key words ‘PI3K/AKT’ and ‘PI3K/AKT/mTOR’ were searched for and genes with a relevance score >4.0 were selected.

### Gene Expression Profiling Interactive Analysis (GEPIA)

**Figure 1:**
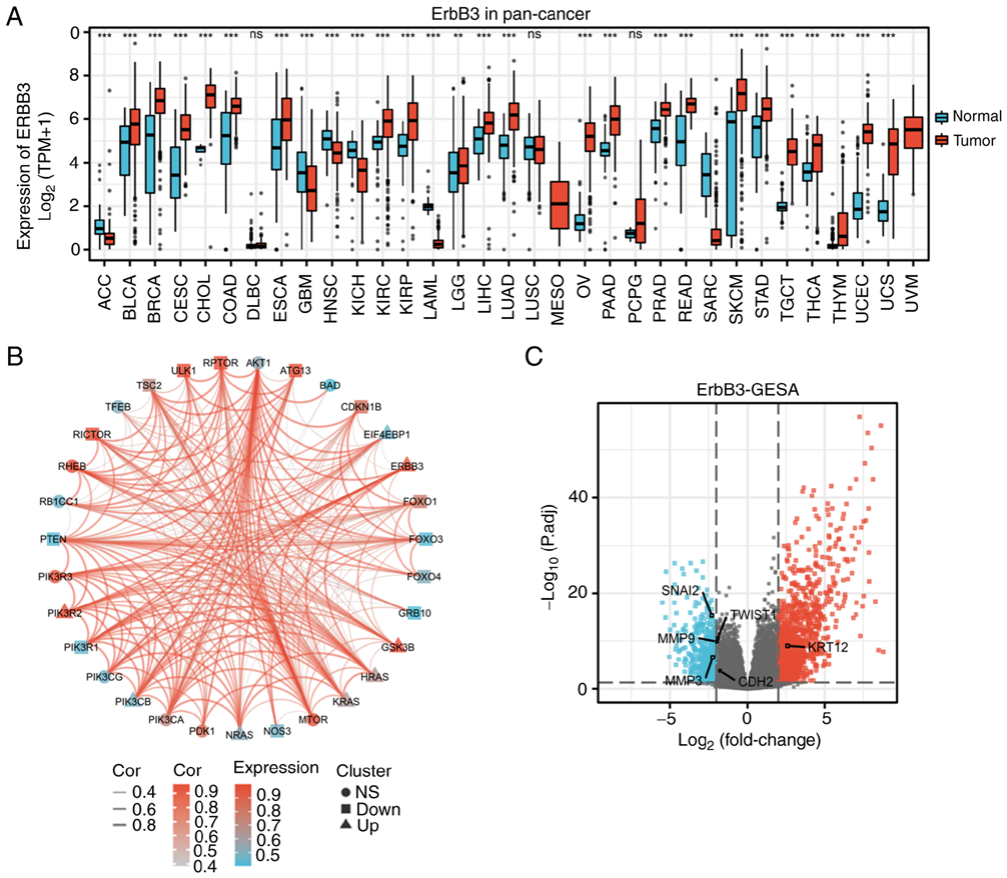
ERBB3 multiomic analysis. (A) RNA-seq TPM of tumor tissue and normal tissue in in different types of cancer to demonstrate ERBB3 (ENSG00000065361) expression (Wilcoxon rank-sum test; ns, not significant; ^**^P<0.01; ^***^P<0.001). (B) Circle-curve correlation diagram of the ERBB3 and 30 genes related to the PI3K/AKT/mTOR pathway in CESC. The upright triangles represent the highly expressed genes, the inverted triangles represent the poorly expressed genes and the circles represent genes that have no significantly different expression in cervical cancer. (C) Volcano map of differentially-expressed genes, where red represents the upregulated genes and light blue represents the downregulated genes. Seq, sequencing; ERBB3; TPM, transcripts per million reads; ns, not significant; CESC, cervical squamous cell carcinoma and adenocarcinoma.

GEPIA (http://gepia.cancer-pku.cn/index.html) is a user-friendly web portal for gene expression analysis based on TCGA and GTEx data. In the current study, expression analysis of ERBB3 was evaluated using the project ID of TCGA-CESC. In the module ‘Expression DIY’ of GEPIA, the expression of ERBB3 between pan-cancer and normal tissue samples was studied with the option of matching normal TCGA data and GTEx data (Fig. 1A).

### Differentially-expressed genes (DEGs)

Batch correction, normalization and difference analysis of RNA-seq data from GSE63514, GSE9750, and GSE44001 were performed to screen for DEGs in CESC samples. GSE63514(22), GSE9750(23) and GSE44001 (24,25) were obtained from the NCBI Gene Expression Omnibus database (ncbi.nlm.nih.gov/geo). The GSE63514 dataset used the GPL570 [HG-U133 Plus 2] Affymetrix Human Genome U133 Plus 2.0 Array platform, which contained 28 cervical cancer tissue samples and 24 normal samples. The GSE9750 dataset used the GPL96 Affymetrix Human Genome U133A Array platform and included 33 cervical cancer tissue samples that were primarily marked by HPV16 or HPV18, and 21 normal cervical samples. The GSE44001 dataset used the GPL14951 Illumina HumanHT-12 WG-DASL V4.0 R2 expression beadchip, which contained 300 cervical cancer tissue samples. GSE63514 and GSE9750 were set as the reference group and GSE44001 as the test group, and the R software limma package was used to identify DEGs between the groups (26). A total of 13,473 DEGs, including 6,514 downregulated and 6,595 upregulated genes, were identified in cervical cancer. The results were visualized using R software (version 3.6.3) (statistical analysis and visualization) with the R package ggplot2 [version 3.3.3] (27) to generate a volcano plot (Fig. 1C), which identified important genes.

### Genomic alteration types and alteration frequency analysis

Genomic alteration types (missense mutation with putative driver or unknown significance, amplification and no alterations) and alteration frequency of 14 EMT-associated genes and 30 PI3K/AKT/mTOR pathway-associated genes were obtained from the cBioPortal for Cancer Genomics (http://www.cbioportal.org), using the ‘OncoPrint’ module and ‘Cancer Types Summary’ module for visualization.

### Immune cell infiltration estimation

For the immune infiltration analysis, transcriptome or other omics data was used to calculate the score of immune cells in the tissue through algorithms, and inferred the infiltration of immune cells in the tissue. Single-sample Gene Set Enrichment Analysis (GSEA) in immune infiltration, which uses the markers of each type of immune cells (28), was used as the gene set to calculate the enrichment of each type of immune cells in each sample.

### Gene Ontology (GO) Term and Kyoto Encyclopedia of Genes and Genomes (KEGG) pathway enrichment analysis and GSEA

GO and KEGG (29,30) analyses were applied to explore the biological functions of target genes in CESC. GO analysis is a powerful bioinformatics tool to determine the biological processes (BPs), cellular components (CCs) and molecular functions (MFs) related to ERBB3. GSEA was utilized to investigate the potential mechanisms of ERBB3. GO, KEGG and GSEA were performed using the R (version 3.6.4) package ClusterProfiler (31). P<0.1 and q<0.2 were selected as the cut-off level.

### Protein interactions and biological processes

The direct and indirect relationship between ERBB3 and 30 hub genes in the PI3K/AKT/mTOR signaling pathway were analyzed using the online tool STRING (https://string-db.org).

### Reverse transcription-quantitative PCR (RT-qPCR)

The MecDNA-HUtrC007Ce01 commercial chip (cat. no. 8*R100-M-20190104; Shanghai Outdo Biotech Co., Ltd.) contains cDNA reverse transcribed from RNA extracted from six cervical cancer cell lines: CaSki, MS751, ME180, C33A, SiHa and HeLa. According to the manufacturer's instructions, qPCR was performed using SYBR^®^ Premix Ex Taq™ II (Tli RNaseH Plus; RR820Q,; Takara Bio, Inc.). Briefly, following the addition of 20 µl qPCR MasterMix into each well, the Axigen PlateMax Ultraclear Sealing Film (UC-500) was used to seal the chip, and it was placed on ice for 15 min to fully dissolve the freeze-dried cDNA. The chip was then centrifuged at 1,750 x g for 3 min at a temperature ramping rate of 2˚C/sec. qPCR was performed using a Roche LightCycler^®^ 480II (Roche Diagnostics) with the following program: Initial denaturation (95˚C, 30 sec); 40 cycles of denaturation (95˚C, 5 sec), annealing (60˚C, 30 sec) and elongation (95˚C, 5 sec); final elongation (60˚C, 1 min) and a final hold (60˚C). The fold-change of gene expression was calculated using 2-(ΔCq experimental group-ΔCq control group) (32). β-actin was used as an internal control and primers are as follows: ERBB3 Forward, 5'-GACCCAGGTCTACGATGGGAA-3'; ERBB3 reverse, 5'-GTGAGCTGAGTCAAGCGAG-3'; human β-actin forward, 5'-GAAGAGCTACGAGCTGCCTGA-3'; human β-actin reverse, 5'-CAGACAGCACTGTGTTGGCG-3' (product length, 191 bp).

### Risk survival analysis

Kaplan-Meier curves (33) can describe the survival status of each group of patients or the survival status of each group of experimental animals. The present study analyzed RNA-seq data from TCGA-CESC cohort (n=304) and selected the median as the cutoff value. Hazard ratio (HR) is defined as the ratio of the two risk rates. When HR is >1, the research object is a risk factor; when HR is <1, the research object is a protective factor; when HR=1, the research object has no effect on survival time. As an outcome index, overall survival (OS) refers to the time to death. The prognostic data come from a Cell article (34). Data filtering: control/normal (not all items have control/normal) were removed and clinical information was retained. For the. nomogram chart, on the basis of multifactor regression analysis, the ruler score was set to characterize the situation of each variable in the multifactor regression model, and finally the total score was calculated to predict the probability of event occurrence (35).

### Transcriptomics analysis

The key regulators in the PI3K/AKT/mTOR pathway were searched using TRRUST (https://www.grnpedia.org/trrust/), a reliable, intuitive tool for human and mouse transcriptional regulatory networks (36).

### Statistical analysis

Software R (version 3.6.3) (37) was used for statistical analysis and visualization. For differential analysis of single gene expression, the R package of ggplot2 (version 3.3.3) (38) was used for visualization. For multigene association analysis, we used the R package of igraph (version 1.2.6) (39) and ggraph package (version 2.0.5) (40). For GO-KEGG analysis and GSEA, the R package of ggplot2 and cluster Profiler package was used (33). Visualization of Kaplan-Meier OS analysis was based on the use of the R package of survminer (0.4.9 version) (41) and for statistical analysis of survival data the survival package (3.2-10 version) was used. Wilcoxon rank sum test was used to assess differences in gene expression. Spearman's rank correlation coefficient was used to assess the significance of correlations. The qPCR data are presented as the mean ± standard deviation of three experiments, and qPCR and RNA-seq data were analyzed using one-way ANOVA followed by Tukey's multiple comparison test. P<0.05 was considered to indicate a statistically significant difference.

## Results

### Genomics

*ERBB3 single-gene DEG in CESC.* In the CESC group, the average level of the normal group was 3.598±1.642, while the average level of the tumor group was 5.539±0.902. The difference was statistically significant (P<0.001) (Fig. 1A). Closely associated genes to ERBB3 in the PI3K/AKT/mTOR pathway were PIK3CA, PIK3R2, PIK3R3, ATG13, MTOR, RICTOR, RHEB and GSK3B (Fig. 1B).

**Figure 2:**
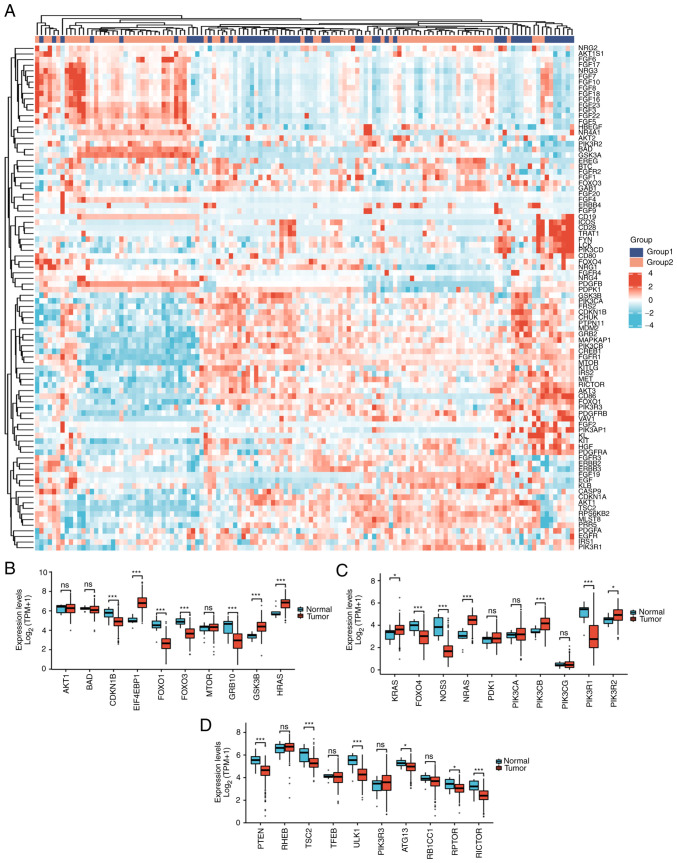
(A) Heat map of the expression levels of 89 PI3K/AKT signaling-related genes in CESC differentially-expressed genes (group 1 as the reference group for GSE63514 and GSE9750, and group 2 as the test group for GSE44001). (B) Differential gene expression between tumor and normal tissues for 10 genes in the PI3K/AKT/mTOR signaling pathway. (C) Differential gene expression between tumor and normal tissues for 10 genes in the PI3K/AKT/mTOR signaling pathway. (D) Differential gene expression between tumor and normal tissues for 10 genes in the PI3K/AKT/mTOR signaling pathway. *P<0.05 and ***P<0.001. ns, not significant; CESC, cervical squamous cell carcinoma and adenocarcinoma. For visualization purposes, (B-D) display a total of 30 genes in the PI3K/AKT/mTOR signaling pathway.

The total number of gene identifications (IDs) after removing the null value was 35,905. Among them, 1,706 IDs met the |log2(FC)|>2 and P<0.05 threshold. Under this threshold, there were 1,221 IDs with high expression (positive logFC) and 485 with low expression (negative logFC). The genes that met this threshold and were significantly associated with EMT included MMP3 and SNAI2 (downregulated genes), and KRT12 (upregulated gene) (Fig. 1C). The expression of seven hub genes in the PI3K/AKT/mTOR pathway were increased in cervical cancer: EIF4EBP1, GSK3B, HRAS, KRAS, NRAS, PIK3CB and PIK3R2 (Fig. 2).

**Figure 3:**
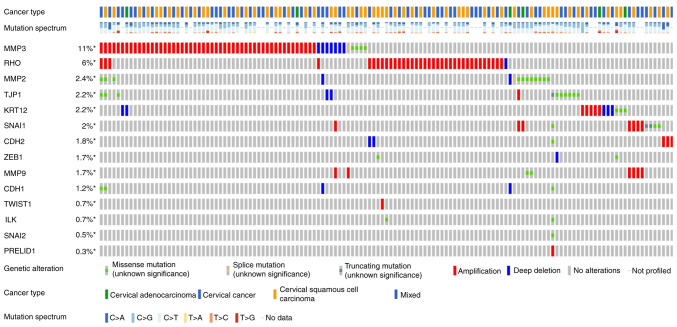
Genomic alteration types and alteration frequency of 14 EMT-associated genes in CESC were analyzed through the ‘OncoPrint’ module and ‘Cancer Types Summary’ module. CDH1, E-cadherin; KRT12, cytokeratin 12; TJP1, tight junction protein 1; CDH2, N-cadherin; VIM, vimentin; ZEB1, zinc finger E-box-binding homeobox 1; ILK, integrin-linked protein kinase.

**Figure 4:**
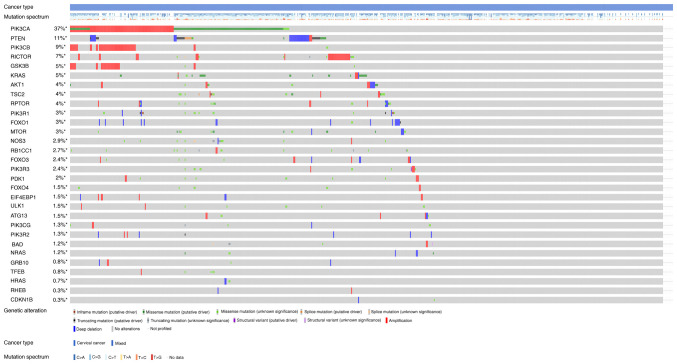
Genomic alteration types and alteration frequency of 30 PI3K/AKT/mTOR pathway-associated genes in CESC were analyzed through the ‘OncoPrint’ module and ‘Cancer Types Summary’ module.

Figs. 3 and 4 show the somatic variation pattern in cervical cancer. These schematics represented the distribution of the number of protein-altering somatic mutations and copy number variations in 607 samples of cervical cancer. The highest frequencies of mutations among the EMT-related genes were revealed in MMP3 (11%, including amplification, deep deletion and missense mutation); PIK3CA (37%, including amplification and missense mutation); and PTEN (11%, including deep deletion and truncating mutation). PIK3CA mutation status assesses the hotspot mutations of the PIK3CA gene (42).

### Selection of EMT-related genes with GO and KEGG analyses

Among 30 PI3K/AKT/mTOR pathway-related genes, upregulated genes in cervical cancer were as follows: EIF4EBP1 (P<0.001), GSK3B (P<0.001), HRAS (P<0.001), KRAS (P<0.05), NRAS (P<0.001), PIK3CB (P<0.001), and PIK3R2 (P<0.001); downregulated genes included CDKN1B (P<0.001), FOXO1 (P<0.001), FOXO3 (P<0.001), GRB10 (P<0.001), FOXO4 (P<0.001), NOS3 (P<0.001), PIK3R1 (P<0.001), PTEN (P<0.001), TSC2 (P<0.001), ULK1 (P<0.001), ATG13 (P<0.05), RPTOR (P<0.05) and RICTOR (P<0.001). Therefore, it is of certain significance to study this pathway in relation to carcinogenesis and prognosis of cervical cancer (Fig. 2B-D).

**Figure 5:**
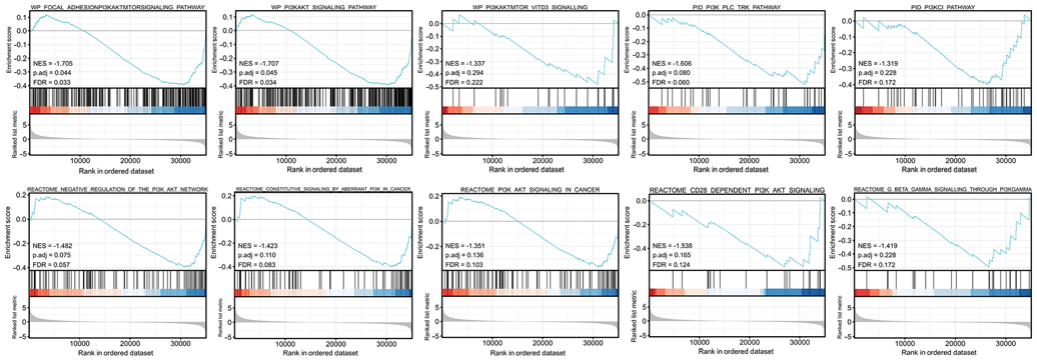
Gene set enrichment analysis of ERBB3. The present study considered that the threshold of significant enrichment was as follows: FDR <0.25, NES <0 and P<0.05. FDR, false-discovery rate; NES, normalized enrichment score.

It was revealed that in cervical cancer the ERBB3 gene was enriched in the PI3K/AKT signaling pathway (NES=-1.707, P=0.045, FDR=0.034; Fig. 5).

**Figure 6:**
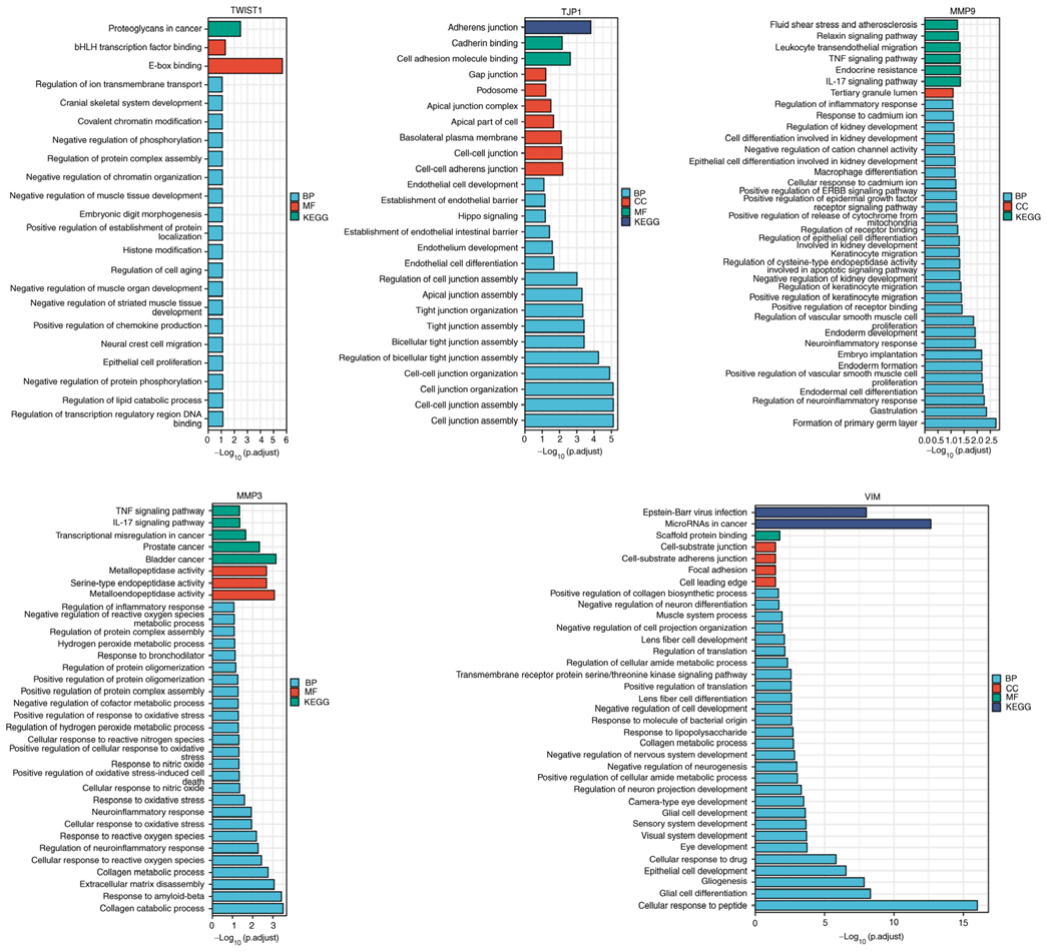
GO-KEGG cluster analysis. The x-axis represents ‘-log(adj. P)’; the greater the value, the stronger the significance. The y-axis represents the GO term. Each color represents an enrichment, including BP, CC, MF and KEGG. KEGG, Kyoto Encyclopedia of Genes and Genomes; GO, Gene Ontology; BP, biological processes; CC, cellular components; MF, molecular functions; TJP1, tight junction protein 1; VIM, vimentin.

TWIST1, tight junction protein 1 (TJP1), MMP9, MMP3 and vimentin (VIM) (Fig. 6) with all annotated functional molecules were compared using hypergeometric distribution tests to determine which functional roles were involved in that stack. The function of genes was divided into three categories: BPs, CCs and MFs.

**Figure 7:**
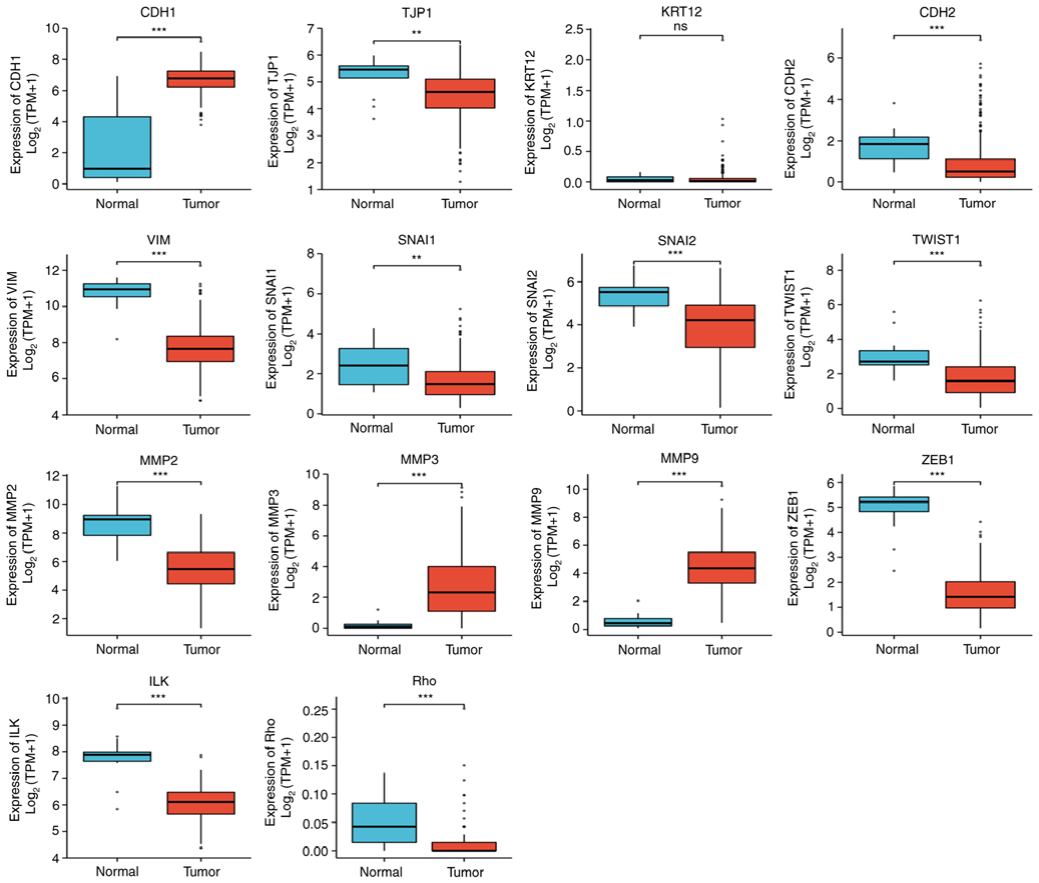
The following significant differences in the expression of epithelial-mesenchymal transition related-factors in CESC were found: CDH1↑, CDH2↓, VIM↑, SNAI2↓, TWIST1↑, MMP2↓, MMP3↑, MMP9↑, ZEB1↓, ILK↓, RHO↓, TJP1↓ and Snail1↓ (statistical method, Wilcoxon rank-sum test). ^**^P<0.01 and ^***^P<0.001. Ns, not significant; CDH1, E-cadherin; KRT12, cytokeratin 12; TJP1, tight junction protein 1; CDH2, N-cadherin; VIM, vimentin; ZEB1, zinc finger E-box-binding homeobox 1; ILK, integrin-linked protein kinase.

**Figure 8:**
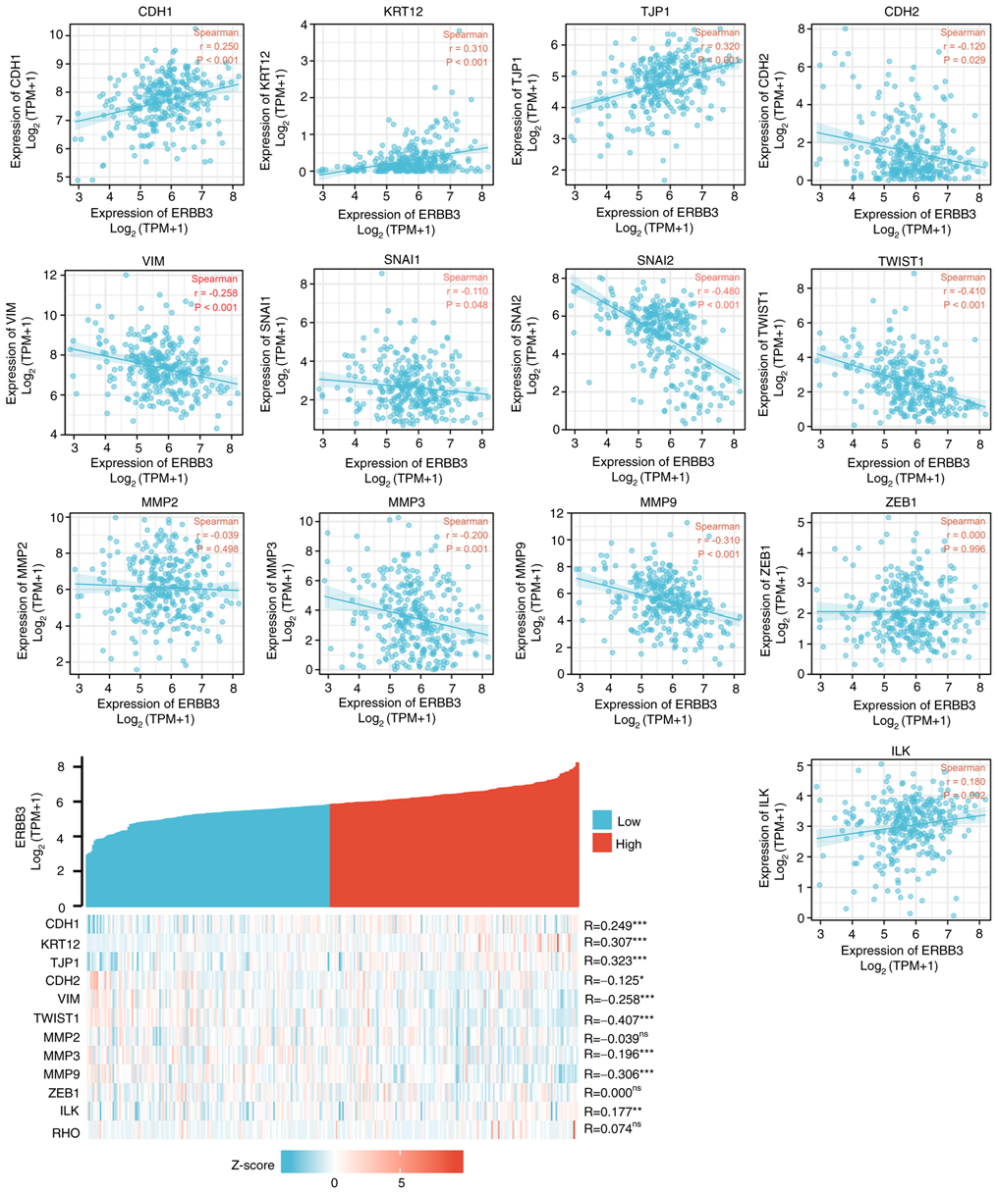
Correlation between ERBB3 and factors associated with epithelial-mesenchymal transition status. Statistically significant were TWIST1 (R=-0.407; P<0.001), TJP1 (R=0.323; P<0.001), KRT12 (R=0.307; P<0.001), MMP9 (R=-0.306; P<0.001). Statistical method, Spearman's correlation; ^*^P<0.05, ^**^P<0.01 and ^***^P<0.001. ns, not significant; CDH1, E-cadherin; KRT12, cytokeratin 12; TJP1, tight junction protein 1; CDH2, N-cadherin; VIM, vimentin; ZEB1, zinc finger E-box-binding homeobox 1; ILK, integrin-linked protein kinase.

Significant differences in EMT-related gene expression in cervical cancer were revealed. The following gene expression levels were upregulated: E-cadherin (CDH1), VIM, TWIST1, MMP3 and MMP9. The following gene expression levels were downregulated: N-cadherin (CDH2), SNAI2, MMP2, zinc finger E-box-binding homeobox 1 (ZEB1), integrin-linked protein kinase (ILK), RHO, TJP1 and SNAIL1 (Fig. 7). Further study on the correlation degree of ERBB3- and EMT-related factors revealed the following results: R=0.307, P<0.001 for KRT12; R=0.323, P<0.001 for TJP1; R=-0.407, P<0.001 for TWSIT1; and R=-0.306, P<0.001 for MMP9 (Fig. 8).

### Transcriptomics

The key regulators in the PI3K/AKT/mTOR pathway are as follows: AKT1, BAD, CDKN1B, FOXO1, FOXO3, FOXO4, GSK3B, HRAS, KRAS, NOS3, NRAS, PDK1, PIK3CA, PIK3R3, PTEN, RPTOR, TSC2 and ULK1. It was revealed that nine transcription factors (TSC22D3, TP53, AR, RELA, NFKB1, STAT3, PPARG, SP1 and JUN) were associated with the regulation of the PI3K/AKT/mTOR pathway (Table I).

### Proteomics

**Figure 9:**
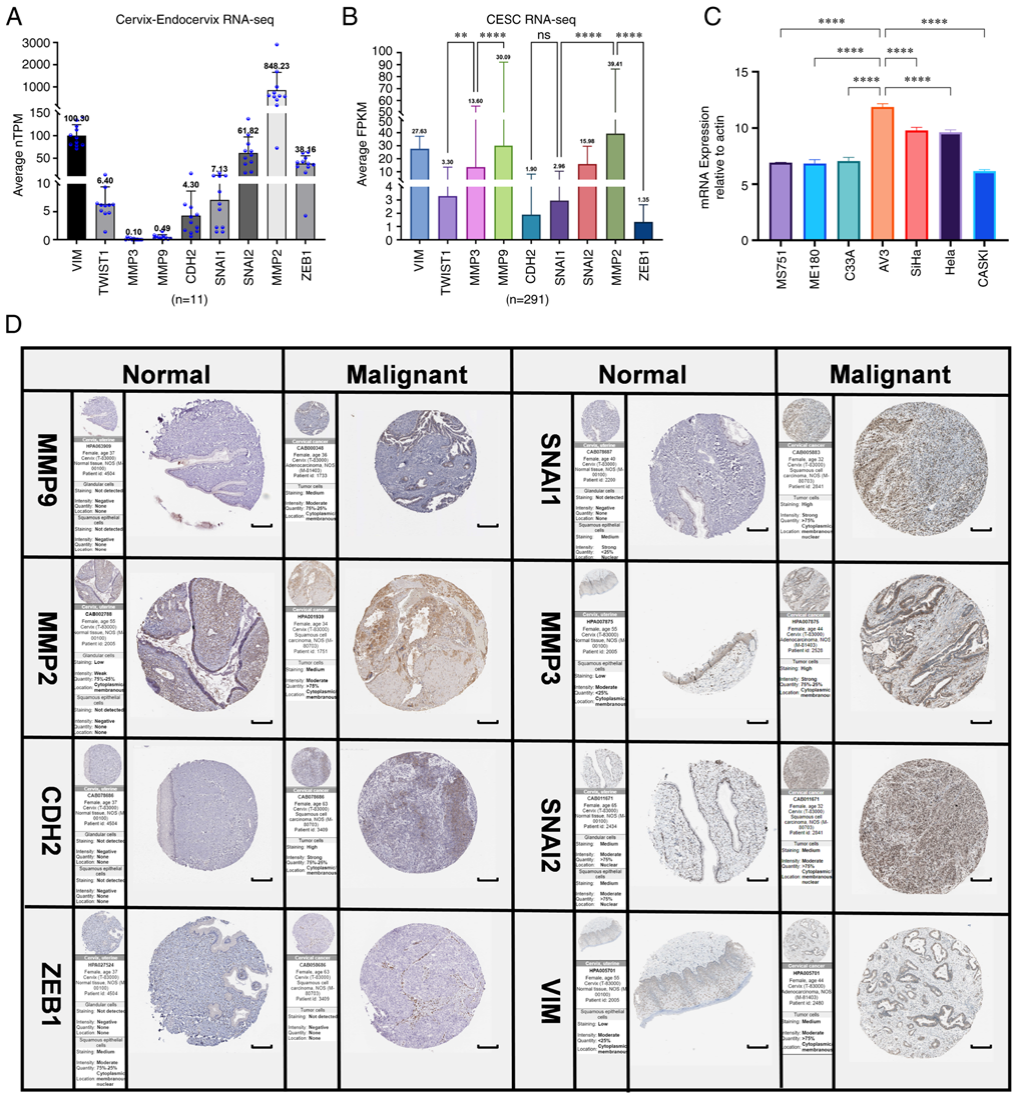
RNA-sequencing and protein level analysis of EMT upregulated factors (PRELI domain-containing protein 1, CDH2, TWIST1, MMP2, MMP3, MMP9, SNAI1, SNAI2 and ZEB1) in normal cervical epithelium and tumor tissues by immunohistochemistry for cervical cancer invasiveness assessment. (A) Normalized TPM was quantified, as shown. The dot plot depicts the means and standard deviation of 11 images of normal cervix-endocervix tissues. (B) FPKM was quantified of 291 images of cervical cancer tissues. (C) ERBB3 expression in seven different cell lines were examined using reverse transcription-quantitative PCR. All PCR data were calculated relative to β-actin and represent the average ± SD of triplicate samples. (D) Immunohistochemical validation of the most significant EMT-related genes in cervical cancer and normal cervix tissues by The Human Protein Atlas database (scale bars, 200 µm). The translational expression level (mRNA and protein) of the eight EMT-related genes was positively correlated with disease status as they were upregulated in cervical squamous cell carcinoma and adenocarcinoma samples. ANOVA followed by Tukey's multiple-comparison tests; ^**^P<0.01 and ^****^P<0.0001. EMT, epithelial-mesenchymal transition; CDH1, E-cadherin; KRT12, cytokeratin 12; TJP1, tight junction protein 1; CDH2, N-cadherin; VIM, vimentin; ZEB1, zinc finger E-box-binding homeobox 1; ILK, integrin-linked protein kinase; FPKM, fragments per kilobase per million; ERBB3, Erb-B2 receptor tyrosine kinase 3; TPM, transcripts per million.

**Figure 10:**
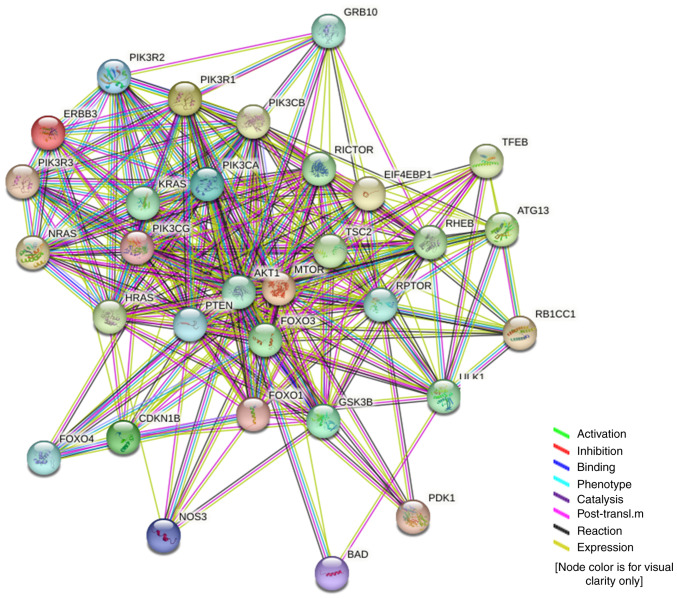
STRING interaction network in the PI3K/AKT/mTOR signaling pathway to show the protein-protein interaction with ERBB3.

By comparing with the RNA level in normal cervical epithelium (Fig. 9A), it was revealed that the expression level of MMP9 in cervical cancer was significantly different. Through IHC analysis of the mRNA-protein expression of the EMT-related genes in cervical cancer tissues, it was revealed that the expression level of MMP family was relatively higher compared with those of other EMT-related genes (Fig. 9B). EMT of cervical cancer is associated with upregulation of MMP9. According to our previous findings of ERBB3 sequencing of cervical cancer cell lines (43), the mRNA levels of different primary cervical cancer cell lines indicated that ERBB3 is highly expressed in cervical malignant cell lines dominated by SiHa and HeLa (Fig 9C). After further research on pathological types and HPV typing, it was revealed that in the three types of cells with higher expression of ERBB3, there was no significant difference between SiHa and HeLa, while there was a significant difference between SiHa and C33A (P<0.0001). These data show that ERBB3 is closely associated with adenocarcinoma and HPV-positive cervical carcinoma. Fig. 10 shows the protein interactions of the 30 key factors in the PI3K/AKT/mTOR signaling pathway with ERBB3. The color of the lines in the figure shows that ERBB3 may play a stimulating role in the PI3K/AKT/mTOR pathway.

### EMT status and immuno-oncology insights from the RNA-seq-based analyses. Immune microenvironment characteristics of CESC

**Figure 11:**
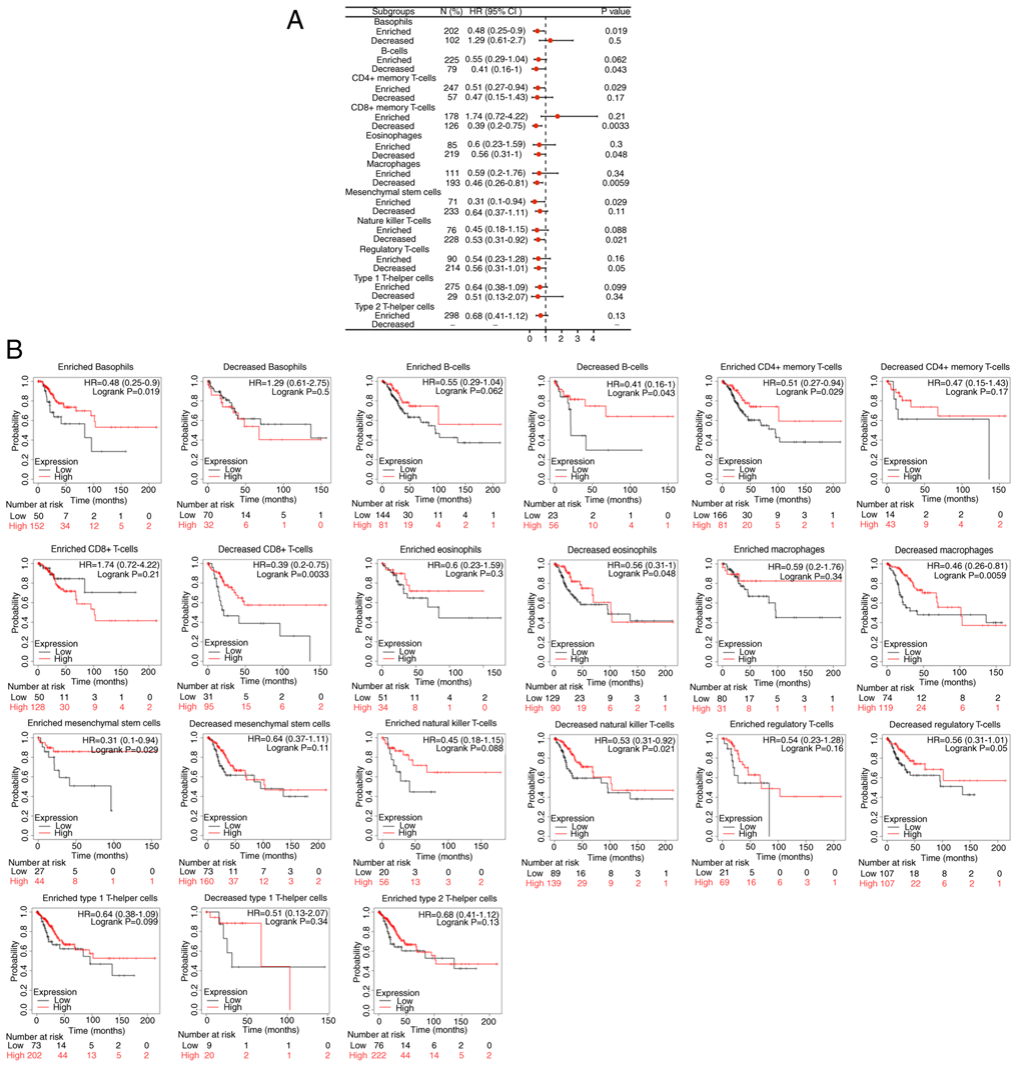
Kaplan-Meier survival curves according to high and low expression of ERBB3 in immune cell subgroups in CESC. (A) A forest plot shows the six prognostic values of ERBB3 expression according to different immune cell subgroups in patients with CESC. (B) Associations between ERBB3 expression and overall survival in different immune cell subgroups in patients with CESC were estimated using Kaplan-Meier plotter. CESC, cervical squamous cell carcinoma and adenocarcinoma samples; HR, hazard ratios.

ERBB3 influenced the survival time of patients with CESC, partially through immune cell infiltration. Enriched basophils (P<0.05), decreased B cells (P<0.05), enriched CD4^+^ memory T cells (P<0.05), enriched mesenchymal stem cells (P<0.05), decreased eosinophils (P<0.05), decreased natural-killer T cells (P<0.05), decreased CD8^+^ memory T cells (P<0.01) and decreased macrophages (P<0.01). Among the abovementioned eight types of immune cell infiltration, the prognosis of the group with a higher ERBB3 mRNA level was decreased (Fig. 11).

### EMT-related genes change the immune cell infiltration

**Figure 12:**
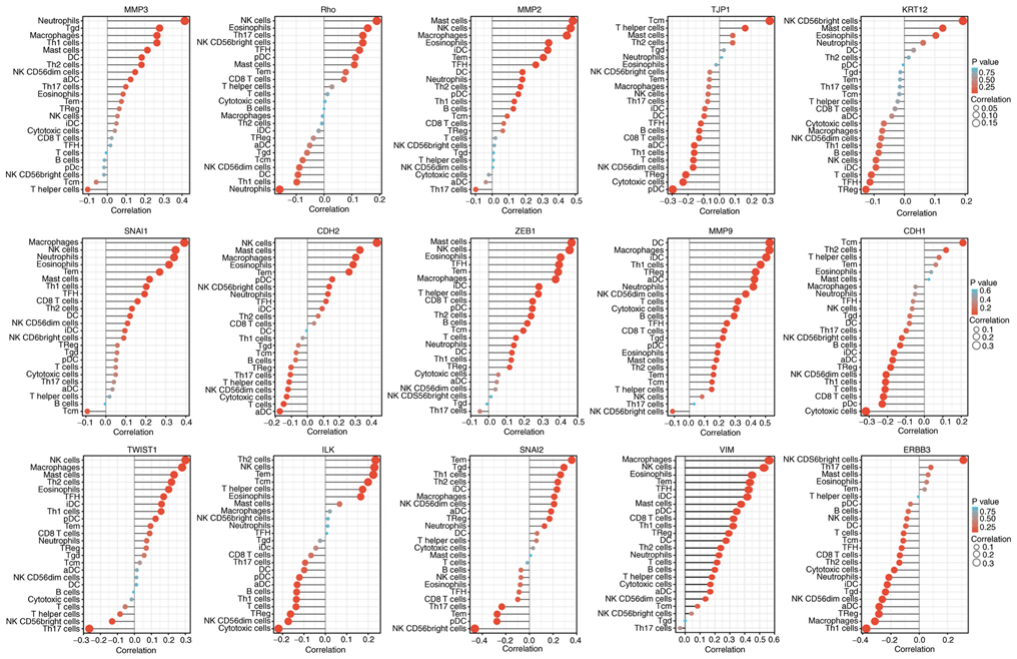
Single sample GSEA enrichment set the score to infer the infiltration by immune cells in each sample. The size of the circle represents the degree of relevance. The greater the height of the bar (the distance from 0), the higher the degree of correlation (a positive number represents a positive correlation, and a negative number represents a negative correlation). The depth of the circle represents the P-value obtained by the correlation analysis, the legend on the right is the color scale value, and the range of the color scale is automatically generated according to the range of the P-value obtained in the figure. CDH1, E-cadherin; KRT12, cytokeratin 12; TJP1, tight junction protein 1; CDH2, N-cadherin; VIM, vimentin; ZEB1, zinc finger E-box-binding homeobox 1; ILK, integrin-linked protein kinase.

If the absolute value of R is below 0.3, there is no straight phase off; R of ≥0.3 denotes linear correlations; R values between 0.3 and 0.5 indicate low-degree correlations; R values between 0.5 and 0.8 refer to significant correlations; and R values of 0.8 and above are high-degree correlations. As shown in Table II, correlation between the EMT-related factors and immune cell infiltration was not high. Among the EMT-related factors, MMP9, MMP2, and ZEB1 were closely associated with the immune system. The EMT status (Fig. 12) may be related to MMP9 changing the tumor immune microenvironment through dendritic cells and macrophages (10).

### Prognostic analysis of microenvironment phenotypes

**Figure 13:**
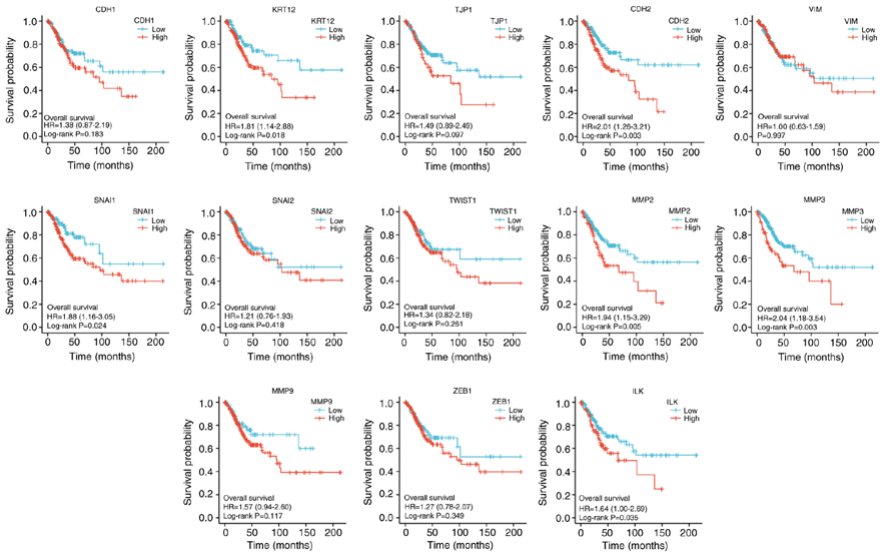
Kaplan-Meier overall survival curves according to high and low expression of CDH1, KRT12, TJP1, CDH2,VIM, SNAI1, SNAI2, TWIST1, MMP2, MMP3, MMP9, ZEB1, and ILK in cervical squamous cell carcinoma and adenocarcinoma samples tumor immune microenvironment. CDH1, E-cadherin; KRT12, cytokeratin 12; TJP1, tight junction protein 1; CDH2, N-cadherin; VIM, vimentin; ZEB1, zinc finger E-box-binding homeobox 1; ILK, integrin-linked protein kinase; HR, hazard ratio.

Statistically significant EMT-related genes that can predict the OS index of CESC included KRT12 (P<0.05), VIM (P<0.05), SNAI1 (P<0.05), ILK (P<0.05), CDH2 (P<0.01), MMP2 (P<0.01) and MMP3 (P<0.01). All of the seven factors were the risk factors for CESC (Fig. 13).

**Figure 14:**
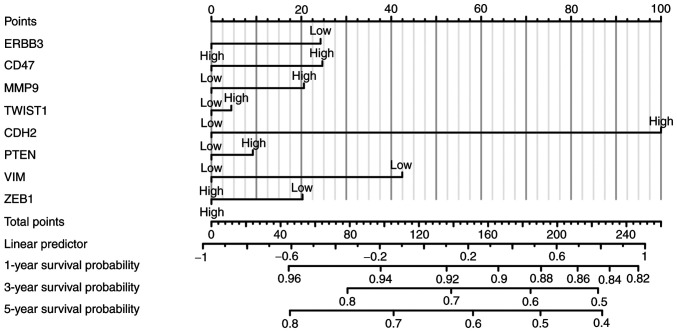
Multivariate model (nomogram chart of Cox regression) of cervical squamous cell carcinoma and adenocarcinoma for progression-free interval. CDH2, N-cadherin; VIM, vimentin; ZEB1, zinc finger E-box-binding homeobox 1.

Risk score was constructed using eight selected genes through the multifactor analysis of the disease prognosis model, and three prognostic types of OS, disease-specific survival and progression-free interval (PFI) were analyzed. ERBB3, CD47, MMP9, TWIST1, CDH2, PTEN, VIM and ZEB1 were included. Multivariate analysis revealed that CDH2, MMP9, and VIM were significant factors in the assessment of PFI. Gene signatures of cervical cancer cell immune-oncology microenvironment positively correlated with the patients' survival. These analyses (Fig. 14) indicated that the selected genes and constructed risk prognostic models had good prognostic value.

## Discussion

High-risk HPV16 DNA is integrated into the host cell genome (HPV16: q21-q31 of chromosome 2.13; HPV18: chromosome 24.8) (44), disrupts the open reading frame, and causes overexpression of E6 and E7 genes (45). It has been demonstrated that E6 and E7 exert carcinogenic effects by combining with cell cycle regulators, such as p53 (a transcription factor related to the PI3K pathway as shown in Table I) and retinoblastoma (46). E6 can interact with E6-related protein E6AP to form a complex and bind to p53(47), hydrolyze p53, and cause the loss of negative regulation of cell proliferation induced by p53, thereby leading to uncontrolled cell proliferation and malignant transformation. The present study also revealed that ERBB3 was highly expressed in HPV-infected cell lines and was associated with adenocarcinoma. Therefore, it can be speculated that HPV-positive cervical cancer cells and adenocarcinoma are the carcinogenic factors or prognostic factors of cervical cancer.

To investigate the association between the actual activation of the PI3K pathway and immune infiltration, the DEGs of ERBB3 in CESC was assessed (GSE63514, GSE9750 and GSE44001 datasets from the Gene Expression Omnibus database were analyzed) for hub genes of the PI3K/AKT/mTOR pathway and cancer progression. Pathway enrichment, protein-protein interaction and pathway crosstalk analyses were performed to identify key genes and pathways. The current study illustrated that cancer-immune interactions might differ depending on specific alterations in the PI3K pathway, demonstrating that genetic aberrations in malignant cells influence the immune landscape of tumors.

The diversity of EMT creates a wide range of heterogeneity in tumors, and may provide tumor cells with increased adaptability and resistance, enabling them to survive and proliferate in a complex TME, and metastasize and invade lymph and blood vessels. The present study demonstrated that MMPs, especially MMP9 as a prominent representative, are highly relevant for TME and immune cells. MMPs are a family of zinc-dependent endopeptidases (48,49). The biological function of MMPs is to degrade various molecules used for cell adhesion and regulate the interaction between cells and the extracellular matrix. Recent studies (50-52) have shown that MMPs are highly associated with the microenvironment of tumors and immune cells, and targeting MMPs may overcome the barriers of immunosuppression. However, the present study revealed that the expression of MMP9 was not a significant predictor of OS in patients with cervical cancer. MMP9 was significantly associated with ERBB3.

In the complex TME, the same anti-infection immune cells can be destroyed by tumor cells (53). As a result, the antitumor immune cells not only do not destroy the transformed cells, but they even change to immune cells that promote tumor growth and metastasis (54,55). These immune cells secrete factors that promote survival, promote migration, and resist detection. Hence, the present study discussed the mechanism of accelerating cervical cancer tumor progression from the perspective of EMT-associated immune evasion.

Cellular immunity is necessary for clearing HPV-infected and HPV-transformed tumor cells. HPV-specific CD8 cytotoxic T lymphocytes (CTLs) are needed for the immune defense against cervical cancer. However, the function of CTLs may be blunted by systemic and local immunosuppressive environments associated with tumor growth (56,57). A series of clinical trials (58-60) have shown that the immune system is unable to completely eradicate the tumor despite the presence of HPV-specific T cells in HPV-associated neoplastic tissue, which suggests the possible existence of systemic immunosuppression and an immunosuppressive TME that significantly influence the efficacy of therapeutic vaccines.

Tumor-associated macrophages (TAMs) may cause the disruptive change of antitumor immunity in TME and promote tumor growth and metastasis (61). TAMs are a heterogeneous population of cells that display a range of phenotypes depending on the type of tumor and their location in TME (62,63). TAMs are commonly the most abundant infiltrating leukocytes in most tumors and are predominantly thought to have pro-tumor effects. These include both immunosuppressive effects in addition to pro-antigenic and metastatic effects. TAMs also promote tumor immune evasion through expression of signal regulatory protein α (SIRPα) (64,65). SIRPα is a receptor for CD47(66), a cell surface protein that typically protects normal cells from phagocytosis by macrophages or dendritic cells. CD47 is frequently overexpressed on tumor cells and plays a key role in tumor escape by binding to SIRPα and sending macrophages a ‘don't eat me’ signal (67,68). Blockade of the CD47-SIRPα signal has been shown to stimulate phagocytosis, leading to tumor cell elimination (69).

In the present study, TAMs were revealed to drive tumor angiogenesis and progression in a spontaneous model of cervical cancer through the production of MMP-9. Previous studies (60,70) showed that MMP9 alone did not significantly affect the survival rate of cervical cancer; however, in the present study, when TME macrophages decreased, there was an impact on OS, and the OS with high ERBB3 was significantly reduced. The GO and KEGG enrichment of MMP9 demonstrated that it participated in the biological process of the IL-17 signaling pathway. In patients with bullous pemphigoid, monocyte-derived macrophages, but not lymphocytes, respond to CXCL10 (upregulated by IL-17) by increasing MMP-9 release, potentially creating an inflammatory loop associated with disease outcome (71).

Late after pattern recognition receptors stimulation, bone-marrow-derived dendritic cells (BMDCs) induce the glucose transporter GLUT1 (72,73) and commit to aerobic glycolysis via the mTOR-HIF-1α/iNOS axis (74), which generates NO, inhibiting the electron transport chain. This process might decrease expression of MHC and co-stimulatory molecules by activated BMDCs (75).

Intratumoral immune cells are more often present in tumors with increasing PI3K downstream phosphorylation (76-78). This is most pronounced for MMP9-positive cells. Research data shows that disturbances in the PI3K pathway may help immune escape (79). A prospective trial in cervical cancer suggested that PI3K pathway alterations may be associated with the composition of TME (80,81).

Previous studies (82-84) have suggested that when cells undergo EMT and shift progressively from an epithelial to a mesenchymal state, genetic alterations include decreased expression levels of CDH1, cytokeratin 12 and TJP1, increased expression levels of CDH2, VIM, SNAI1, SNAI2, TWIST1, MMP2, MMP3, MMP-9 and ZEB1 and increased activity of ILK and RHO.

It can be inferred from the present study that tumor infiltration by CD8-positive lymphocytes was associated with PIK3CA mutations and worse clinical outcome. At the molecular level, EMT transcriptional factors, including SNAl, ZEB1 and TWIST1, regulated immunosuppressive cells or enhanced the expression of immunosuppressive checkpoint molecules through the production of chemokines, thereby resulting in immunosuppression of TME. Immunosuppressive factors can also induce EMT in tumor cells. This mutual feedback between EMT and immunosuppression promotes tumor progression (85).

**Figure 15:**
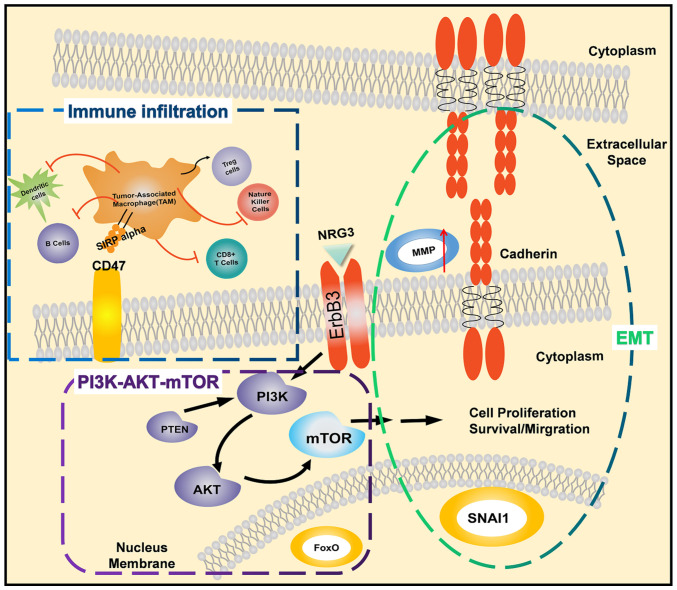
ERBB3-PI3K/AKT/mTOR pathway mediates the change of epithelial-mesenchymal transition status of cancer cells and contributes to the tumor immune microenvironment.

In conclusion, integrating the characteristics of biomarkers in multiple dimensions can ensure the most efficient management choice for each patient with cancer (Fig. 15). The EMT status cannot be assessed based on one or a few molecular markers, but should be assessed in conjunction with changes in cell characteristics to assess the current ability of cell metastasis and distant invasion. Immuno-oncology research can generate the discriminating power and richness of data required for these features. In particular, simple MMP changes are not a prognostic factor in cervical cancer. When ERBB3 activates the PI3K pathway to change immune cell infiltration, the cervical cancer prognosis model is meaningful.

## Figures and Tables

**Table I:** Key transcription factors of PI3K/AKT/mTOR biomarkers in cervical squamous cell carcinoma and adenocarcinoma.

Key transcription factor	Description	Regulated gene	P-value	FDR
TSC22D3	TSC22 domain family, member 3	FOXO4, FOXO3, FOXO1	3.62x10^-8^	3.26x10^-7^
TP53	Tumor protein p53	HRAS, PTEN, CDKN1B, AKT1, FOXO3	5.57x10^-6^	2.51x10^-5^
AR	Androgen receptor	AKT1, TSC2, NRAS, PTEN	1.37x10^-5^	4.12x10^-5^
RELA	V-rel reticuloendotheliosis viral oncogene homolog A (avian)	PTEN, BAD, FOXO3, NOS3, AKT1	1.02x10^-4^	1.90x10^-4^
NFKB1	Nuclear factor of kappa light polypeptide gene enhancer in B-cells 1	PTEN, AKT1, GSK3B, FOXO3, NOS3	1.06x10^-4^	1.90x10^-4^
STAT3	Signal transducer and activator of transcription 3 (acute-phase response factor)	PTEN, CDKN1B, AKT1	1.46x10^-3^	2.19x10^-3^
PPARG	Peroxisome proliferator-activated receptor gamma	BAD, PTEN	4.92x10^-3^	6.33x10^-3^
SP1	Sp1 transcription factor	CDKN1B, PTEN, NOS3, HRAS	6.32x10^-3^	7.11x10^-3^
JUN	Jun proto-oncogene	PDK1, NOS3	2.33x10^-2^	2.33x10^-2^

FOXO, Forkhead box; CDKN1B, cyclin-dependent kinase inhibitor 1B; TSC2, tuberous sclerosis 2; BAD, Bcl2-associated agonist of cell death; NOS3, nitric oxide synthase 3; GSK3B, Glycogen synthase kinase-3 β; PDK1, pyruvate dehydrogenase (acetyl-transferring) kinase isozyme 1.

**Table II:** ERBB3 methylation and immune infiltration in tumor microenvironment of cervical cancer.

Cell type	CDH1	CDH2	ERBB3	ILK	KRT12	MMP2	MMP3	MMP9	PRELID1	RHO	SNAI2	SNAI1	TJP1	TWIST1	ZEB1
aDC	-0.172^a^	-0.173^a^	-0.283^b^	-0.132	-0.041	-0.038	0.124^c^	0.427^b,d^	0.028	-0.051	0.183^a^	0.033	-0.159^a^	0.015	0.046
B cells	-0.131^c^	-0.074	-0.078	-0.134	-0.085	0.127^c^	-0.016	0.293^b^	0.014	-0.000	-0.072	-0.004	-0.128^c^	-0.005	0.217^b^
CD8 T cells	-0.222^c^	0.042	-0.137^c^	-0.067	-0.031	0.069	0.022	0.228^b^	0.056	0.072	-0.099	0.159^a^	-0.129^c^	0.090	0.247^b^
Cytotoxic cells	-0.318^c,d^	-0.130^c^	-0.177^a^	-0.219^b^	-0.068	-0.021	0.040	0.306^b,d^	0.077	0.003	0.031	0.049	-0.231^b^	-0.017	0.054
DC	-0.081	-0.005	-0.095	-0.099	0.030	0.182^a^	0.184^a^	0.527^c,e^	0.064	-0.094	0.065	0.121^c^	-0.097	0.011	0.131^c^
Eosinophils	0.037	0.285^b^	0.052	0.165^a^	0.103	0.339^b,d^	0.084	0.184^a^	0.009	0.157^a^	-0.078	0.316^b,d^	-0.022	0.202^b^	0.404^b,d^
iDC	-0.165^a^	0.092	-0.226^b^	-0.045	-0.094	0.334^b,d^	0.048	0.505^b,e^	0.006	-0.019	0.233^b^	0.095	-0.092	0.160^a^	0.283^b^
Macrophages	-0.048	0.301^b^	-0.313^b,d^	0.021	-0.073	0.449^b,d^	0.266^b^	0.526^c,e^	0.142^c^	-0.005	0.210^b^	0.393^b,d^	-0.067	0.281^b^	0.376^b,d^
Mast cells	0.025	0.328^b,d^	0.062	0.066	0.126^c^	0.485^b,d^	0.214^b^	0.177^a^	-0.108	0.109	0.011	0.219^b^	0.084	0.234^b^	0.466^b,d^
Neutrophils	-0.050	0.128^c^	-0.214^b^	0.011	0.062	0.181^a^	0.415^b,d^	0.420^b,d^	0.112	-0.160^a^	0.127^a^	0.341^b^	0.015	0.072	0.142^c^
NK CD56 bright cells	-0.123^a^	0.136^c^	0.312^b,d^	0.012	0.193^b^	0.013	-0.019	-0.111	-0.081	0.140^c^	-0.463^b,d^	0.089	-0.061	-0.131^c^	0.014
NK CD56dim cells	-0.208^b^	-0.122^c^	-0.261^b^	-0.174^a^	-0.076	0.004	0.149^a^	0.370^c,d^	0.028	-0.089	0.208^b^	0.108	-0.167^a^	0.012	0.039
NK cells	-0.066	0.434^b,d^	-0.088	0.229^b^	-0.094	0.471^b,d^	0.054	0.083	0.043	0.190^b^	-0.072	0.349^b^	-0.072	0.301^b^	0.455^b,d^
pDC	-0.229^c^	0.155^a^	-0.062	-0.121	-0.004	0.157^a^	-0.018	0.188^b^	0.070	0.113^c^	-0.275^b^	0.051	-0.296^b^	0.124^c^	0.245^b^
T cells	-0.213^c^	-0.148^a^	-0.111	-0.137	-0.109	0.020	-0.007	0.319^b,d^	0.052^a^	0.012	-0.018	0.049	-0.165^a^	-0.055	0.153^a^
T helper cells	0.079	-0.114^c^	-0.006	0.174^a^	-0.022	0.006	-0.106	0.146^c^	-0.015	0.029	0.059	0.019	0.162^a^	-0.085	0.279^b^
TCM	0.209^b^	-0.070	-0.113^c^	0.199^b^	-0.017	0.091	-0.060	0.148^a^	-0.182	-0.077	0.358^b,d^	-0.093	0.318^b,d^	0.032	0.195^b^
TEM	0.062	0.260^b^	0.038	0.224^b^	-0.014	0.307^b,d^	0.075	0.157^a^	0.101	0.078	-0.273^b^	0.269^b^	-0.064	0.095	0.391^b,d^
TFH	-0.061	0.116^c^	-0.124^c^	0.007	-0.113^c^	0.262^b^	0.017	0.246^b^	0.032	0.128^c^	-0.088	0.194^b^	-0.118^c^	0.172^a^	0.396^b,d^
TGD	-0.079	-0.057	-0.239^b^	-0.025	-0.013	0.007	0.280^b^	0.219^b^	-0.011	-0.061	0.292^b^	0.055	0.028	0.057	-0.010
Th1 cells	-0.211^b^	-0.030	-0.374^b,d^	-0.136	-0.082	0.133^c^	0.265^b^	0.468^b,d^	-0.014	-0.098	0.263^b^	0.202^b^	-0.160^a^	0.157^a^	0.127^c^
Th17 cells	-0.099	-0.106	0.082	-0.095	-0.015	-0.099	0.099	0.031	0.136^c^	0.140^c^	-0.234^b^	0.040	-0.075	-0.266^b^	-0.049
Th2 cells	0.118^c^	0.067	-0.140^c^	0.235^b^	0.014	0.170^a^	0.183^a^	0.162^a^	-0.043	-0.007	0.240^b^	0.130^c^	0.083	0.218^b^	0.239^b^
T Reg	-0.183^a^	-0.103	-0.285^b^	-0.163^a^	-0.127^c^	0.064	0.063	0.435	0.033	-0.038	0.170^a^	0.058	-0.213^b^	0.070	0.118^c^

The significance of correlations between immune cell infiltration in cervical cancer tissue and the expression of different genes was determined using Spearman's rank correlation coefficient.

^a^P<0.01,

^b^P<0.001 and

^c^P<0.05;

^d^moderate and

^e^high correlation. CDH, cadherin; ILK, integrin-linked protein kinase; KRT12, cytokeratin 12; PRELID1, PRELI domain-containing 1; TJP1, tight junction protein 1; ZEB1, zinc finger E-box-binding homeobox 1; DC, dendritic cells; NK, natural killer cells; Th, T helper; aDC, activated DC; iDC, immature DC; pDC, plasmacytoid DC; TCM, T central memory; TEM, T effector memory; TFH, T follicular helper; TGD, T γδ.

## Data Availability

The datasets generated and analyzed during the current study are available in the cervical squamous cell carcinoma and endocervical adenocarcinoma (CESC) data in TCGA (https://portal.gdc.cancer.gov/). The datasets used and/or analyzed during the current study are available from the corresponding author on reasonable request.
